# Undergraduate teaching in family medicine within the PRIMAFAMED network

**DOI:** 10.4102/phcfm.v16i1.4376

**Published:** 2024-01-30

**Authors:** Robert Mash, Innocent Besigye, Anna Galle

**Affiliations:** 1 Division of Family Medicine and Primary Care, Faculty of Medicine and Health Sciences, Stellenbosch University, Cape Town, South Africa; 2 Department of Family Medicine, School of Medicine, Makerere University, Kampala, Uganda; 3 Department of Public Health and Primary Care, Faculty of Medicine and Health Sciences, University of Ghent, Ghent, Belgium

## Introduction

The Primary Care and Family Medicine (PRIMAFAMED) network connects departments of family medicine and primary care from academic institutions across sub-Saharan Africa.^1^ Currently, there are 20 countries and approximately 40 institutions in the network.^2^ The network has a focus on educational and research capacity building with a South-South-North philosophy. There is, therefore, a strong emphasis on collaboration within the region with support from strategic partners, such as Ghent University and the University of Bergen.

The 14th meeting of the network took place in Johannesburg, South Africa on 15 August 2023–16 August 2023. One of the sessions at the meeting was on undergraduate education. Each participant was asked to prepare an interactive poster. They were asked to share: * what is happening with family medicine education for medical students at your institution, and in particular to reflect on the strengths and weaknesses of what is happening. What innovations or lessons might others learn from your educational programme?*

Each participant also sent a 250 word abstract on their poster. At the meeting, each participant had 5 min to 10 min to present their poster and discuss it with a group of other participants. The authors conducted a thematic analysis of the abstracts, posters, and notes taken during the session to create the conference report presented here.

## Current state of undergraduate teaching

### Exposure to family medicine and primary health care

Exposure to family medicine and primary health care (PHC) varied considerably, from some programmes with no clinical exposure (i.e. Aga Khan University in Kenya and Tanzania, Amoud University in Somaliland) to 52 weeks in the new curriculum at Stellenbosch University, South Africa (Table 1). The majority of programmes had short clinical rotations in years 4–6. In a few cases, these exposures were dominated by public health (e.g. Ethiopia, Zimbabwe). In several universities, the exposure was planned to increase as the curriculum was revised. A few programmes made an attempt to balance exposure to both rural and urban practice as well as PHC and district hospitals. In Nigeria, the exposure was often in the tertiary hospital (i.e. general outpatients, emergency unit) and private family practice. Undergraduate exposure to family medicine was seen as instrumental in increasing awareness and understanding of the discipline, and in motivating people to consider this as career choice.

**TABLE 1:** Exposure to family medicine in undergraduate programmes in sub-Saharan Africa.

Country	Institution	Exposure to family medicine teaching	Weeks of clinical exposure	Exposure to family medicine clinical training
Somaliland	Amoud University	Family physicians contribute extensively to the general curriculum	0	None, previously 4 weeks in 6th year
South Africa	University of Cape Town	18-months ‘Becoming a doctor’ course in years 2–3; 6-week ‘Health in context’ integrated course in year 4	5	1 week in 4th year and 4 weeks in 6th year (20% of students have longitudinal rural clerkship)
Kenya	Moi University	PBL across years 1 to 6; CDM/palliative care in year 6	30	COBES across years 1 to 5
Nigeria	Bowen University	Didactic lectures on family medicine principles in years 4–5	14	Case-based discussions and PBL at the bedside in tertiary hospital and in community, direct observation of procedural skills
South Africa	Stellenbosch University	Teaching in all modules years 1–3	32–52	Exposure to PHC every 2 weeks in years 1–3; final year is a distributed apprenticeship in DHS.
Ethiopia	Addis Ababa University	Teaching on palliative care and observership	6	Community medicine for 6 weeks (PH and FM)
Malawi	Kamuzu University of Health Sciences	Teaching week on FM.	8–14	6-week rotation in FM (becoming 8-weeks); 4-weeks in DH in 4th year (becoming 6 weeks)
Botswana	University of Botswana	Years 1–3 then year-5	16	Exposure in years 1–2 to urban PHC, then clinical rotation 8-weeks in years 3 and 5.
Namibia	University of Namibia	Teaching during years 2, 3, 4 and 6.	8	4-weeks in years 2, 3 and 4 in rural community
Zimbabwe	University of Zimbabwe	Lectures in year 3 in PH	0	Short community attachment in PH (new curriculum plans more FM)
DRC	Protestant University of Congo	20 h of theory in PHC and 15 h for FM.	4	4-weeks practical training in PHC in year 1 as a holiday placement
Zambia	University of Zambia	None	8	8-week clerkship
Tanzania	Aga Khan University	None	0	None
Kenya	Aga Khan University	Teaching in other modules in years 1–6	0	None
South Africa	University of Limpopo	Teaching in modules in years 1–3, 5	22	7-weeks PHC in year 3, 9-weeks PHC in year 5, 6-weeks DH in year 6.
Uganda	Makerere University	Lectures for 1-week in 4th year	4	4-weeks clinical placement

PBL, problem based learning; CDM, chronic disease management; COBES, community based education and service; PHC, primary health care; DHS, district health services; PH, public health; FM, family medicine; DH, district hospital; DRC, Democratic Republic of Congo.

### Availability of family medicine teachers and trainers

A common theme was a lack of family physicians or people with postgraduate training in family medicine to train and teach students. The ratio of students to competent trainers was an issue in several universities, particularly those with larger student numbers. Having registrars at the postgraduate level could increase the pool of trainers. Sometimes, general practitioners and medical officers, with no background in family medicine, performed training.

### Educational approach

The emphasis was on clinical training within PHC or sometimes district hospitals. In these settings, learning revolved around actual patients and their specific problems, often described as ‘bedside teaching’, demonstration and practice of skills, ward rounds, tutorials, as well as case-based discussions. Students learnt clinical decision making, procedural skills and became more familiar with the communities served and their health challenges.

In a few instances, the university emphasised peer learning and participatory group or pair learning. This could be a strategy to cope with large student numbers. Moi University emphasised that their approach was ‘community-based and community-orientated’, and involved an investigative project on the community. In several cases, the exposure to PHC integrated inputs from family medicine and public health, and sometimes other disciplines, such as rehabilitation. This integration brought together a focus on community-oriented primary care for patients, with a focus on public health for the whole community and social determinants of health. Many programmes still had lectures prior to the clinical exposure to teach theory, often in the ‘pre-clinical’ years.

In Malawi, they decided to include teaching on planetary health following the cyclone that devastated the country. Several programmes also included a focus on palliative care that was linked to family medicine. One programme had introduced a specific focus on continuity of care.

There was a general principle that teaching and exposure to family medicine and PHC should start as early as possible. Stellenbosch University, in their new curriculum from 2022, exposes all students in years 1–3 to the same PHC facility every 2-weeks. In years 1–2, the students are facilitated by nurse practitioners, which also promotes inter-professional respect and learning.

Innovative approaches to learning were used to cope with the trainer-student ratios and multiple sites. These might include digital solutions such as ‘virtual consultations’ at the University of Cape Town. Digital solutions were also catalysed during the coronavirus disease 2019 (COVID-19) pandemic and the ‘creation of a virtual learning platform’ at Bowen University. Namibia has introduced an electronic portfolio of learning.

Both Stellenbosch University and the University of Cape Town were promoting a longitudinal spiral curriculum where students continually revisited and built on prior learning. This type of curriculum also relied on interdisciplinary and interdepartmental collaboration around common goals, moving away from the ‘professional tribalism’ that rotates students in silos between separate departments and disciplines.

Mirroring this approach to learning, assessment was also moving towards being more programmatic, continuous and workplace-based. This might involve portfolios of learning, that included group projects, written assignments or observations, using tools such as the Mini-Clinical Evaluation Exercise for Trainees (miniCEX) or Direct Observation of Procedural Skills (DOPS). Stellenbosch University and University of Namibia were also using the Assessment of Brief Behaviour Change Counselling (ABC) tool.^3^ Having separate, summative examinations in family medicine could be burdensome, and more integrated examinations might just include a component from family medicine. Some programmes, however, continued to offer a final family medicine examination with Multiple Choice Questions (MCQs), Objective Structured Clinical Examinations (OSCEs), and oral examinations.

### Teaching in other modules and disciplines

Family physicians were versatile teachers by virtue of their generalist background. In Somaliland, they were the most available specialists and taught much of the curriculum. Family physicians might contribute to the learning of consultation and communication skills, population health management, medical ethics, emergency medicine, health systems, leadership, and clinical governance.

As family medicine develops, it is sometimes housed in public or community health departments. While the integration of primary care and public health approaches was potentially beneficial, it was also important for family medicine to have governance over its own programmes.

### Community-based teaching and learning

Learning in PHC, district hospitals and communities prepared students for internship, helped them develop approaches to undifferentiated problems, and gave them a better understanding of the district health system outside the teaching hospital. Home visits were mentioned as a way of presenting students with ‘disorientating dilemmas’ that stimulated reflection and dialogue on family and contextual issues.

However, community-based teaching and learning was noted to be cost and labour intensive as students must travel, sometimes be accommodated in rural settings and could only be placed in smaller groups across multiple facilities. In addition, it was harder to standardise teaching and training quality across facilities. Primary health care was sometimes a poor learning environment because of the absence of ‘equipment, drugs and other resources’ and poor infrastructure.

## Conclusions

When compared with a previous evaluation within the PRIMAFAMED network, it appears that exposure to PHC and family medicine has increased at several universities (e.g. Moi, Malawi, Stellenbosch).^4^ The undergraduate footprint is still very heterogeneous with some institutions having no exposure and others increasing exposure substantially through curriculum renewal.

There is a clear linkage between the success of under- and post-graduate training programmes. Undergraduate programmes need clinical trainers with postgraduate training, while recruitment in postgraduate programmes is partly dependent on exposure at an undergraduate level. The availability of clinical trainers with family medicine and educational expertise remains an issue. The learning environment can also be problematic as the PHC level of the health system is often neglected and under-resourced. Upskilling medical officers and general practitioners through shorter diplomas may be a useful strategy.^5^

Several members of the PRIMAFAMED network have the intention to create a College of Family Medicine for East, Central and Southern Africa, which could facilitate specialist training in these countries.^6^ Strengthening postgraduate training should lead to better prepared clinical trainers at the undergraduate level.

Community-based training is beneficial to the developing doctor and may prepare them better for internship and the health needs of the population. Students are exposed to undifferentiated problems, the relevance of a more holistic approach, can learn to be more community-orientated, and understand how the whole health system functions. On the other hand, community-based training requires additional financial resources, which is difficult in resource-constrained environments. In some settings, safety may also be an issue in the community. This shift requires political and academic commitment to the benefits of community-based training in PHC and family medicine, and the shifting of teaching resources from tertiary hospitals to more distributed platforms.

Feedback from participants pointed towards some innovative approaches and future directions. There appears to be a shift towards more programmatic and workplace-based assessment, which is also happening in postgraduate education.^7^ There is also a shift to more integrated, multidisciplinary, summative assessments. The use of digital technology to support community-based training is also emerging and will most likely increase in the future. Integration of primary care and public health approaches should be retained, even as family medicine becomes more mature as a separate discipline. Efforts to map the education of family medicine within the region via the PRIMAFAMED network should continue, as this can lead to further exchange of best practices, enhanced collaboration, and pooling of resources. The PRIMAFAMED conference and the publication of this article was supported by a Short Initiative grant from the Flemish Interuniversity Council (VLIR) (Reference ZA2022SIN355A103).
